# Age and sex-specific associations of anthropometric measures of adiposity with blood pressure and hypertension in India: a cross-sectional study

**DOI:** 10.1186/s12872-016-0424-y

**Published:** 2016-12-01

**Authors:** Kevin Y. Taing, Michael E. Farkouh, Rahim Moineddin, Jack V. Tu, Prabhat Jha

**Affiliations:** 1 Centre for Global Health Research, St. Michael’s Hospital, Toronto, ON Canada; 2 Dalla Lana School of Public Health, University of Toronto, Toronto, ON Canada; 3 Peter Munk Cardiac Centre, University Health Network, Toronto, ON Canada; 4 The Heart and Stroke Richard Lewar Centre of Excellence in Cardiovascular Research, University of Toronto, Toronto, ON Canada; 5 Department of Family and Community Medicine, University of Toronto, Toronto, ON Canada; 6 Schulich Heart Centre, Sunnybrook Health Sciences Centre, Toronto, ON Canada; 7 Institute for Clinical Evaluative Sciences, Toronto, ON Canada; 8 Institute of Health Policy, Management and Evaluation, University of Toronto, Toronto, ON Canada

**Keywords:** Age, Sex, Anthropometry, Body mass index, Waist circumference, Adiposity, Blood pressure, Hypertension, India

## Abstract

**Background:**

A determinant of blood pressure is adiposity; however, there are uncertainties surrounding whether general or central adiposity is the more important determinant of blood pressure. Further, inconsistent results exist for the relationships of anthropometric measures with blood pressure and hypertension, and whether these relationships differ substantially by age and sex is unclear. We aimed to elucidate the associations of anthropometric measures of general and central adiposity with blood pressure and hypertension, and determine the effect of age and sex on these relationships.

**Methods:**

We used cross-sectional data from the Centre for Global Health Research health check-up survey conducted during 2006–2007 of the general population in India (*n* = 7 601; age 18–59 years). We examined the associations of anthropometric measures (body mass index, waist circumference, hip circumference, waist-hip ratio, waist-height ratio) with blood pressure components (systolic pressure, diastolic pressure, pulse pressure, mean arterial pressure, mid-blood pressure) and hypertension within four (18–29 years, 30–39 years, 40–49 years, 50–59 years) age groups, by sex. We adjusted all analyses for education and location, with further adjustments, variously, for either a measure of central (waist circumference) or general (body mass index) adiposity.

**Results:**

On average, every 5 kg/m^2^ greater body mass index or 10 cm wider waist circumference was associated with a 5 and 4 mmHg higher systolic blood pressure, respectively. When considered separately, each anthropometric measure was strongly and positively associated with most blood pressure components in all age groups, and for both sexes. However, with few exceptions, when considered jointly (body mass index adjusted for waist circumference), the associations of body mass index with blood pressure components and hypertension were greatly diminished for both sexes, and particularly in the ≥30 years age groups. By contrast, further adjustment of waist circumference for body mass index did not materially alter the associations of waist circumference with blood pressure components and hypertension.

**Conclusions:**

Our findings indicate that central adiposity, as assessed with anthropometric measures, may be a more important determinant of blood pressure and hypertension than general adiposity for adults in India.

## Background

India is undergoing a rapid health transition with substantial increases in chronic non-communicable diseases, such as cardiovascular disease (CVD) [1, 2], whereby elevated blood pressure and hypertension are well-established risk factors [3–6]. High blood pressure accounts for the greatest proportion of deaths attributed to chronic disease risk factors in India [1], and recent estimates show that about 30% of the population is hypertensive [7]. In order to abate the burden of hypertension and related health outcomes, it is important to understand and establish the relationships between major risk factors for elevated blood pressure. However, studies examining these relationships are often from high income countries, thus the results may not be generalizable to low and middle-income countries (LMIC), such as India. In spite of the need for studies investigating the associations between risk factors for hypertension, there is limited reliable evidence from India and other LMICs.

A main determinant of blood pressure is adiposity [8–10], and various mechanisms have been proposed to link different body fat distributions to blood pressure and risk of hypertension [9, 10]. However, despite the suggested mechanisms, there are uncertainties surrounding whether a general or central distribution of adiposity is more strongly associated with blood pressure. Anthropometric indices are often used as a proxy measure of adiposity. Broadly, these anthropometric measures of adiposity can be considered either a measure of general adiposity, such as the body mass index (BMI), or measures of central adiposity, such as waist circumference (WC), hip circumference (HC), and their ratios, waist-hip ratio (WHR) and waist-height ratio (WHtR). Similar to the uncertainties in proposed mechanisms, there are inconsistencies as to whether anthropometric measures of general or central adiposity are more strongly associated with blood pressure and hypertension [11–14]. In addition, considerable uncertainty exists regarding whether the strength of these associations differs substantially by age and sex.

Reliable assessment of the relationships of anthropometric measures with blood pressure and hypertension in India and other LMICs is of particular importance. Clarification of these associations will contribute additional evidence to aid in facilitating the allocation of resources for public health promotion and prevention of hypertension and CVD. Additionally, by improving our understanding of whether a simple measure of general or central adiposity is more strongly associated with blood pressure and hypertension may help focus screening efforts and risk stratification of clinical populations where the measurement of blood pressure is not feasible. However, most previous studies from India are small, from one region, provide conflicting evidence, and do not fully investigate the effect of age and sex on these relationships [11–14]. Indeed, there is a paucity of research from India that directly examines the association of commonly used anthropometric measures of adiposity with blood pressure and hypertension. Thus, these associations remain inadequately characterized. Furthermore, given the importance and potential differences in prognostic value of distinct blood pressure components [systolic blood pressure (SBP), diastolic blood pressure (DBP), pulse pressure, mean arterial pressure, mid-blood pressure] [4, 5], it is equally important to elucidate the relationships between anthropometric measures and continuous blood pressure components, in addition to hypertension.

We therefore examine the independent and joint association of anthropometric measures of general and central adiposity with various blood pressure components and hypertension, and determine the effect of age and sex on these relationships in a sample of adults from the general population of India.

## Methods

### Study design, setting and participants

We conducted a cross-sectional study investigating the relationships between anthropometric measures of adiposity, blood pressure, and hypertension in India. In order to aid in the characterization of disease and death of individuals, households and communities, the Centre for Global Health Research health check-up survey of the general population of India was conducted during 2006 and 2007. Participants were recruited in four states (Andhra Pradesh, Karnataka, Gujarat, Rajasthan) and two union territories (Chandigarh, Delhi) from randomly selected sampling units (within the Registrar General of India’s “Sample Registration System”), which were based on the 1991 census [15]. We used data from adults 18–59 years of age from the health check-up survey in our analyses (*n* = 7 784). Of these participants, 119 were excluded because of pregnancy, and 64 due to missing data for either level of education, SBP, DBP, height, weight, WC, or HC.

### Study data sources

Field teams consisting of trained surveyors visited houses identified in their respective sampling units to obtain consent and enroll participants (surveyors made at least three visits to each household). All data collection was done in the household following a standardized protocol. In addition to physical measurements, participants were interviewed to obtain information on demographic, socioeconomic, lifestyle characteristics and antihypertensive medication use.

### Blood pressure and anthropometric measurement

Blood pressure was measured twice at heart level, in a seated position after 5 min of rest using the Omron (Kyoto, Japan) digital automatic blood pressure monitor. SBP and DBP were calculated as the average of the two readings, pulse pressure as the difference between SBP and DBP, mean arterial pressure as a third SBP plus two thirds DBP, and mid-blood pressure as half SBP plus half DBP. Hypertension was defined as SBP ≥140 mmHg or DBP ≥90 mmHg, or reported use of antihypertensive medication.

Height was measured with a measuring tape to the nearest 0.1 cm. Individuals were requested to stand upright without footwear, with their head, back, buttocks and heels against a wall, arms at their sides, feet together and eyes directed forward. Height was then measured as the distance from the top of the head to the ground. Weight was measured on a hard level surface to the nearest 0.1 kg using the KRUPS (New Delhi, India) weighing scale, without footwear and only light clothing. WC was measured to the nearest 0.1 cm using a measuring tape. Measurements were taken at the midway point between the lower rib and iliac crest without clothing when possible. If participant did not want to move aside clothing, it was indicated and measurements were taken above light clothing. HC was measured to the nearest 0.1 cm using a measuring tape. Measurements were taken at the point yielding the maximum circumference over the buttocks with individuals wearing light clothing. In order to account for clothing, 1 cm was subtracted from measured values. BMI was calculated as weight in kilograms divided by the square of height in meters. WHR was calculated as WC in centimeters divided by HC in centimeters, and WHtR as WC in centimeters divided by height in centimeters.

### Statistical analysis

We performed all analyses separately for men and women. We calculated Pearson’s partial correlation coefficients for the relationships between anthropometric measures (BMI, WC, HC, WHR, WHtR) and blood pressure (SBP, DBP, pulse pressure, mean arterial pressure, mid-blood pressure), adjusted for age and location. We used multiple linear regression models to quantify the association between each anthropometric measure with each continuous blood pressure component, and Poisson regression models to quantify the relationship between each anthropometric measure and hypertension. We evaluated age as an effect measure modifier by including age and anthropometric measure interaction terms in regression models, and by examining the stratum-specific estimates by age groups (18–29 years, 30–39 years, 40–49 years, and 50–59 years).

We estimated the means of each blood pressure component, and calculated the prevalence ratios (PR) for hypertension per sub-group specific standard deviation (SD) change in anthropometric measures within each age group. 235 individuals (3% of total) who reported current use of antihypertensive medication were excluded from the continuous blood pressure analyses. We used two models for all analyses, model 1 adjusted for level of education (illiterate, primary school, middle school, secondary school, college) and location (11 categories, representing rural/urban area of each state and union territory), and in model 2 additional adjustments were made for either WC or BMI. Further adjustment for alcohol (nondrinker, current drinker) and tobacco (nonuser, current user) use did not materially affect these estimates (results not presented). We performed all statistical analyses using SAS version 9.3 (SAS Institute, Cary, NC, USA), and provide estimates with their respective 95% confidence intervals (CI).

## Results

Distribution of the mean (SD) anthropometric measures, blood pressure components, and hypertension prevalence by sex and age groups are presented in Table 1. The mean age of participants was 40 (11) years. On average, blood pressure increased with age for both sexes, with the exception of DBP, which increased until the fourth decade and remained relatively constant between the fourth and fifth decade. Mean blood pressure (regardless of blood pressure component) was higher for men than women in all age groups. Similarly, hypertension prevalence was higher for men than women, except in the 50–59 years age group where it was comparable.

**Table 1:** Distribution of anthropometric measures and blood pressure (*n* = 7 601)

		BMI (kg/m^2^)		WC (cm)		HC (cm)		WHR		WHtR		SBP (mmHg)		DBP (mmHg)		PP (mmHg)		MAP (mmHg)		MBP (mmHg)		HTN (%)^a^
Age	No.																					
Male																						
18–29	1620	20.6	(3.6)	71.2	(9.8)	84.7	(7.3)	0.84	(0.066)	0.43	(0.058)	123.2	(11.8)	74.7	(9.5)	48.4	(8.8)	90.9	(9.4)	99.0	(9.7)	187 (11.5)
30–39	967	21.9	(4.2)	77.2	(11.8)	87.0	(8.1)	0.88	(0.072)	0.46	(0.067)	124.3	(13.5)	78.8	(10.5)	45.5	(8.2)	94.0	(10.9)	101.6	(11.4)	163 (16.9)
40–49	640	22.7	(4.4)	80.8	(12.4)	88.0	(8.2)	0.92	(0.079)	0.49	(0.071)	129.7	(18.9)	82.5	(12.3)	47.2	(10.9)	98.3	(13.9)	106.1	(15.0)	210 (32.8)
50–59	519	22.7	(4.6)	81.9	(13.4)	87.7	(8.5)	0.93	(0.084)	0.50	(0.078)	133.8	(20.0)	82.6	(11.8)	51.2	(13.0)	99.7	(13.7)	108.2	(15.1)	212 (40.9)
Total	3746	21.6	(4.2)	75.9	(12.2)	86.3	(8.0)	0.88	(0.081)	0.46	(0.072)	126.1	(15.4)	78.2	(11.1)	47.9	(9.9)	94.2	(11.8)	102.1	(12.5)	772 (20.6)
Female																						
18–29	1608	20.4	(4.0)	63.9	(9.2)	84.2	(8.1)	0.76	(0.065)	0.42	(0.060)	112.6	(11.2)	72.7	(8.8)	40.0	(7.5)	86.0	(9.0)	92.7	(9.3)	71 (4.4)
30–39	992	22.5	(4.9)	69.9	(11.5)	88.5	(9.7)	0.79	(0.071)	0.46	(0.075)	116.4	(13.4)	76.5	(9.9)	39.9	(7.7)	89.8	(10.6)	96.4	(11.2)	122 (12.3)
40–49	737	23.8	(5.3)	73.9	(12.3)	90.9	(10.3)	0.81	(0.078)	0.49	(0.079)	123.8	(18.2)	79.9	(10.7)	43.9	(11.5)	94.5	(12.6)	101.8	(13.8)	185 (25.1)
50–59	518	23.8	(5.6)	75.7	(13.3)	91.3	(11.3)	0.83	(0.083)	0.50	(0.087)	132.1	(21.9)	81.6	(11.2)	50.5	(15.5)	98.4	(13.8)	106.8	(15.6)	202 (39.0)
Total	3855	22.0	(4.9)	68.9	(12.0)	87.5	(9.9)	0.78	(0.076)	0.45	(0.079)	118.3	(16.5)	76.2	(10.4)	42.1	(10.5)	90.3	(11.8)	97.3	(12.8)	580 (15.1)

Values presented as mean (SD), unless indicated ^a^No. (%) hypertensive. *BMI* body mass index, *WC* waist circumference, *HC* hip circumference, *WHR* waist-hip ratio, *WHtR* waist-height ratio, *SBP* systolic blood pressure, *DBP* diastolic blood pressure, *PP* pulse pressure, *MAP* mean arterial pressure, *MBP* mid-blood pressure, *HTN* hypertension

The means for all anthropometric measures were lowest in the youngest age group (18–29 years) and increased in older age groups for both men and women. BMI and HC were similar between both sexes in the youngest age group, and slightly higher among women in the older age groups. By contrast, mean WC and WHR were higher (~6–7 cm and 0.08–0.1 respectively) for men than women across all age groups. There were no discernible differences for mean WHtR between the sexes.

The intercorrelations of anthropometric measures and blood pressure components are shown in Table 2. Among the correlations, the weakest were consistently observed between anthropometric measures and pulse pressure.

**Table 2:** Pearson partial correlation coefficients adjusted for age and location (*n* = 7 366)

		WC	HC	WHR	WHtR	SBP	DBP	PP	MAP	MBP
BMI	Male:	0.88	0.84	0.62	0.89	0.27	0.34	0.06	0.33	0.32
	Female:	0.86	0.85	0.47	0.87	0.26	0.34	0.05^*^	0.33	0.31
WC		Male:	0.85	0.82	0.96	0.27	0.35	0.04^*^	0.34	0.33
		Female:	0.82	0.74	0.96	0.27	0.34	0.07	0.33	0.32
HC			Male:	0.40	0.76	0.26	0.32	0.05^*^	0.31	0.30
			Female:	0.24	0.76	0.22	0.31	0.02^**^	0.29	0.27
WHR				Male:	0.84	0.20	0.27	0.01^**^	0.26	0.24
				Female:	0.75	0.21	0.23	0.09	0.23	0.23
WHtR					Male:	0.26	0.34	0.03^**^	0.33	0.31
					Female:	0.28	0.34	0.08	0.34	0.32
SBP						Male:	0.75	0.71	0.91	0.96
						Female:	0.76	0.76	0.92	0.96
DBP							Male:	0.07	0.96	0.91
							Female:	0.14	0.95	0.91
PP								Male:	0.36	0.47
								Female:	0.43	0.54
MAP									Male:	0.99
									Female:	0.99

Correlations exclude those on antihypertensive medication. All correlations coefficients significant at *p* < 0.0001, unless indicated ^*^ *p* < 0.05, ^**^ *p* > 0.05. *BMI* body mass index, *WC* waist circumference, *HC* hip circumference, *WHR* waist-hip ratio, *WHtR* waist-height ratio, *SBP* systolic blood pressure, *DBP* diastolic blood pressure, *PP* pulse pressure, *MAP* mean arterial pressure, *MBP* mid-blood pressure

### Anthropometric measures and continuous blood pressure

The changes in SBP and DBP [mmHg per SD (95% CI)] for each anthropometric measure by sex and age groups are presented in Tables 3 and 4. On average, every 5 kg/m^2^ greater BMI or 10 cm wider WC was associated with a 5 and 4 mmHg higher SBP, and a 4 and 3 mmHg higher DBP, respectively. Independently, each measure was strongly and positively associated with SBP and DBP for both sexes across all age groups (model 1). However, after additional adjustment for WC or BMI, the association of each anthropometric measure with SBP and DBP were markedly different, and largely dependent on the age group (model 2).

**Table 3:** Mean differences in systolic and diastolic blood pressure for anthropometric measures among men (*n* = 3 664)

		mmHg per SD (95% CI)			
		Systolic blood pressure		Diastolic blood pressure	
		Model 1	Model 2	Model 1	Model 2
Measurement (SD)	Age				
Body Mass Index^a^					
(3.6 kg/m^2^)	18–29	4.07 (3.51, 4.62)	2.44 (1.36, 3.53)	3.70 (3.26, 4.14)	1.59 (0.74, 2.45)
(4.2 kg/m^2^)	30–39	4.44 (3.58, 5.31)	2.42 (0.64, 4.20)	3.44 (2.77, 4.10)	0.52 (−0.83, 1.86)
(4.4 kg/m^2^)	40–49	5.39 (3.83, 6.94)	2.11 (−1.39, 5.60)	4.14 (3.15, 5.12)	1.09 (−1.10, 3.28)
(4.6 kg/m^2^)	50–59	4.37 (2.34, 6.40)	0.47 (−3.60, 4.55)	3.50 (2.33, 4.66)	0.89 (−1.44, 3.23)
Waist Circumference^b^					
(9.8 cm)	18–29	3.96 (3.41, 4.51)	1.87 (0.80, 2.95)	3.79 (3.36, 4.22)	2.43 (1.58, 3.28)
(11.8 cm)	30–39	4.39 (3.53, 5.25)	2.29 (0.53, 4.06)	3.75 (3.10, 4.40)	3.30 (1.97, 4.63)
(12.4 cm)	40–49	5.55 (4.00, 7.11)	3.67 (0.17, 7.16)	4.38 (3.40, 5.36)	3.41 (1.21, 5.60)
(13.4 cm)	50–59	4.91 (2.89, 6.93)	4.50 (0.41, 8.59)	3.79 (2.63, 4.95)	3.01 (0.66, 5.35)
Hip Circumference^b^					
(7.3 cm)	18–29	4.03 (3.46, 4.60)	1.87 (0.89, 2.85)	3.62 (3.17, 4.08)	1.59 (0.81, 2.36)
(8.1 cm)	30–39	4.10 (3.21, 4.98)	1.14 (−0.47, 2.74)	3.21 (2.54, 3.89)	1.04 (−0.18, 2.26)
(8.2 cm)	40–49	4.72 (3.12, 6.33)	0.32 (−2.63, 3.28)	3.52 (2.50, 4.54)	−0.10 (−1.96, 1.76)
(8.5 cm)	50–59	4.62 (2.60, 6.64)	3.19 (−0.41, 6.80)	3.70 (2.54, 4.86)	2.56 (0.49, 4.62)
Waist-Hip Ratio^b^					
(0.066)	18–29	2.49 (1.94, 3.04)	0.37 (−0.28, 1.02)	2.54 (2.10, 2.98)	0.75 (0.23, 1.26)
(0.072)	30–39	3.40 (2.55, 4.24)	1.14 (0.05, 2.22)	3.21 (2.58, 3.84)	1.88 (1.06, 2.69)
(0.079)	40–49	4.49 (3.00, 5.98)	1.95 (−0.03, 3.93)	3.74 (2.80, 4.68)	2.03 (0.79, 3.27)
(0.084)	50–59	3.70 (1.77, 5.63)	1.80 (−0.68, 4.28)	2.68 (1.56, 3.80)	0.97 (−0.46, 2.40)
Waist-Height Ratio^b^					
(0.058)	18–29	3.52 (2.97, 4.07)	0.16 (−0.97, 1.28)	3.56 (3.13, 3.99)	1.69 (0.80, 2.57)
(0.067)	30–39	4.25 (3.40, 5.09)	1.97 (0.10, 3.84)	3.63 (2.99, 4.26)	3.22 (1.81, 4.64)
(0.071)	40–49	5.28 (3.76, 6.80)	2.95 (−0.64, 6.55)	4.19 (3.24, 5.14)	3.02 (0.76, 5.28)
(0.078)	50–59	5.19 (3.21, 7.17)	6.44 (2.12, 10.76)	3.78 (2.64, 4.92)	3.38 (0.90, 5.87)

Estimates exclude those on antihypertensive medication. Model 1, estimates from multiple linear regression, adjusted for education and location. Model 2, model 1 plus additional adjustments for ^a^Waist circumference or ^b^Body mass index. *SD* standard deviation, *CI* confidence interval

**Table 4:** Mean differences in systolic and diastolic blood pressure for anthropometric measures among women (*n* = 3 702)

		mmHg per SD (95% CI)			
		Systolic blood pressure		Diastolic blood pressure	
		Model 1	Model 2	Model 1	Model 2
Measurement (SD)	Age				
Body Mass Index^a^					
(4.0 kg/m^2^)	18–29	3.32 (2.78, 3.86)	2.46 (1.44, 3.47)	2.69 (2.27, 3.12)	2.24 (1.45, 3.03)
(4.9 kg/m^2^)	30–39	5.40 (4.55, 6.25)	2.60 (0.85, 4.36)	4.79 (4.18, 5.40)	2.73 (1.48, 3.98)
(5.3 kg/m^2^)	40–49	5.03 (3.65, 6.42)	−0.05 (−2.60, 2.49)	4.26 (3.48, 5.05)	0.79 (−0.65, 2.23)
(5.6 kg/m^2^)	50–59	3.76 (1.70, 5.83)	0.70 (−3.21, 4.62)	2.95 (1.91, 4.00)	1.00 (−0.98, 2.98)
Waist Circumference^b^					
(9.2 cm)	18–29	3.05 (2.52, 3.59)	1.01 (0.01, 2.00)	2.40 (1.97, 2.82)	0.53 (−0.25, 1.31)
(11.5 cm)	30–39	5.47 (4.62, 6.32)	3.19 (1.44, 4.95)	4.74 (4.13, 5.35)	2.35 (1.10, 3.60)
(12.3 cm)	40–49	6.01 (4.64, 7.39)	6.06 (3.50, 8.62)	4.81 (4.04, 5.59)	4.14 (2.69, 5.59)
(13.3 cm)	50–59	4.15 (2.12, 6.19)	3.56 (−0.31, 7.43)	3.12 (2.08, 4.15)	2.28 (0.32, 4.24)
Hip Circumference^b^					
(8.1 cm)	18–29	2.89 (2.33, 3.44)	0.41 (−0.55, 1.37)	2.43 (2.00, 2.86)	0.60 (−0.15, 1.35)
(9.7 cm)	30–39	4.51 (3.65, 5.38)	−0.58 (−2.28, 1.13)	4.07 (3.45, 4.69)	−0.24 (−1.46, 0.98)
(10.3 cm)	40–49	4.69 (3.28, 6.09)	1.07 (−1.83, 3.96)	3.97 (3.17, 4.77)	0.91 (−0.74, 2.55)
(11.3 cm)	50–59	3.01 (1.00, 5.01)	−0.35 (−4.17, 3.47)	2.80 (1.78, 3.81)	1.32 (−0.61, 3.26)
Waist-Hip Ratio^b^					
(0.065)	18–29	1.91 (1.37, 2.45)	0.49 (−0.10, 1.09)	1.33 (0.91, 1.76)	0.12 (−0.34, 0.59)
(0.071)	30–39	4.24 (3.39, 5.09)	2.05 (1.09, 3.02)	3.50 (2.88, 4.13)	1.47 (0.78, 2.15)
(0.078)	40–49	4.95 (3.59, 6.31)	3.44 (1.95, 4.93)	3.64 (2.85, 4.43)	2.22 (1.38, 3.07)
(0.083)	50–59	3.37 (1.38, 5.36)	2.23 (0.04, 4.42)	1.87 (0.84, 2.90)	0.80 (−0.32, 1.91)
Waist-Height Ratio^b^					
(0.060)	18–29	3.06 (2.53, 3.59)	1.08 (0.04, 2.12)	2.45 (2.03, 2.86)	0.72 (−0.09, 1.53)
(0.075)	30–39	5.31 (4.48, 6.14)	3.01 (1.23, 4.80)	4.62 (4.02, 5.21)	2.24 (0.97, 3.51)
(0.079)	40–49	5.76 (4.44, 7.09)	5.75 (3.24, 8.26)	4.53 (3.78, 5.28)	3.62 (2.20, 5.04)
(0.087)	50–59	4.39 (2.39, 6.38)	4.97 (0.92, 9.03)	2.97 (1.96, 3.99)	1.96 (−0.10, 4.03)

Estimates exclude those on antihypertensive medication. Model 1, estimates from multiple linear regression, adjusted for education and location. Model 2, model 1 plus additional adjustments for ^a^Waist circumference or ^b^Body mass index. *SD* standard deviation, *CI* confidence interval

Among men in the 18–29 and 30–39 years age groups, the association between WC and SBP was slightly weaker than the relationship between BMI and SBP. By contrast, the association between WC and DBP was stronger than the relationship between BMI and DBP. Further, in the 30–39 years age group, the association between BMI and DBP was largely diminished (3.44 to 0.52 mmHg) with the additional adjustment for WC. In comparison, additional adjustment for BMI did not materially alter the relation between WC and DBP. Among women in the 18–29 years age group, BMI remained significantly associated with both SBP and DBP after adjusting for WC. However, further adjustment for BMI greatly reduced the strength of association of WC with SBP and DBP, from 3.05 mmHg to 1.01 mmHg and 2.40 mmHg to 0.53 mmHg, respectively.

The additional adjustment for WC greatly attenuated the relationship of BMI with SBP and DBP in the two older age groups (40–49 years, 50–59 years) for both men and women. By contrast, the association of WC with SBP and DBP for men, and WHtR and SBP, and WC and DBP for women were only slightly attenuated and remained significant (*p* < 0.05) after further adjustment for BMI. Overall, the relationship of HC and WHR with SBP and DBP diminished with additional adjustment for BMI for both sexes across all age groups. Comparably, the associations of WHtR with SBP and DBP were also weakened after adjustment for BMI, with the exception of WHtR with SBP in the 50–59 years age group for both sexes.

The analyses of the relationships of anthropometric measures with pulse pressure, mean arterial pressure and mid-blood pressure can be found in Tables 5, 6 and 7. The associations between anthropometric measures and pulse pressure were weaker than those with the other blood pressure components. There was a significant (*p* < 0.05) negative association between WHtR and pulse pressure after adjustment for BMI for men in the 18–29 [−1.53 (−2.43, −0.64)] and 30–39 [−1.25 (−2.45, −0.06)] years age groups (Table 5). This negative association may be due, in part, to the stronger relationship between WHtR and DBP [18–29 years: 1.69 (0.80, 2.57), 30–39 years: 3.22 (1.81, 4.64)], than WHtR and SBP [18–29 years: 0.16 (−0.97, 1.28), 30–39 years: 1.97 (0.10, 3.84)] in those age groups. Greater increases in DBP than SBP per SD higher WHtR would decrease the difference between SBP and DBP, thus lowering pulse pressure. The associations of anthropometric measures with mean arterial and mid-blood pressure were similar to those observed with SBP and DBP, which may be partly due to the high correlations between these blood pressure components. Of note, the relationship between WC and mid-blood pressure was stronger than the association between BMI and mid-blood pressure for men across all age groups, and for women in the ≥30 years age groups.

**Table 5:** Mean differences in pulse pressure for anthropometric measures (*n* = 7 366)

		Male			Female	
		mmHg per SD (95% CI)			mmHg per SD (95% CI)	
		Model 1	Model 2		Model 1	Model 2
Age	Measurement (SD)			Measurement (SD)		
	Body Mass Index^a^			Body Mass Index^a^		
18–29	(3.6 kg/m^2^)	0.37 (−0.07, 0.81)	0.85 (−0.02, 1.72)	(4.0 kg/m^2^)	0.63 (0.25, 1.00)	0.22 (−0.48, 0.92)
30–39	(4.2 kg/m^2^)	1.01 (0.45, 1.56)	1.90 (0.76, 3.04)	(4.9 kg/m^2^)	0.61 (0.08, 1.14)	−0.13 (−1.23, 0.97)
40–49	(4.4 kg/m^2^)	1.25 (0.31, 2.19)	1.02 (−1.09, 3.12)	(5.3 kg/m^2^)	0.77 (−0.13, 1.66)	−0.84 (−2.51, 0.83)
50–59	(4.6 kg/m^2^)	0.87 (−0.48, 2.23)	−0.42 (−3.16, 2.32)	(5.6 kg/m^2^)	0.81 (−0.69, 2.31)	−0.29 (−3.15, 2.56)
	Waist Circumference^b^			Waist Circumference^b^		
18–29	(9.8 cm)	0.17 (−0.27, 0.61)	−0.56 (−1.42, 0.31)	(9.2 cm)	0.66 (0.29, 1.03)	0.48 (−0.22, 1.17)
30–39	(11.8 cm)	0.63 (0.08, 1.19)	−1.01 (−2.14, 0.12)	(11.5 cm)	0.73 (0.20, 1.26)	0.84 (−0.25, 1.94)
40–49	(12.4 cm)	1.17 (0.23, 2.11)	0.26 (−1.85, 2.37)	(12.3 cm)	1.20 (0.30, 2.10)	1.92 (0.24, 3.60)
50–59	(13.4 cm)	1.13 (−0.23, 2.48)	1.49 (−1.25, 4.24)	(13.3 cm)	1.04 (−0.45, 2.52)	1.28 (−1.54, 4.11)
	Hip Circumference^b^			Hip Circumference^b^		
18–29	(7.3 cm)	0.40 (−0.05, 0.86)	0.28 (−0.50, 1.07)	(8.1 cm)	0.46 (0.08, 0.84)	−0.19 (−0.86, 0.47)
30–39	(8.1 cm)	0.88 (0.32, 1.44)	0.10 (−0.93, 1.12)	(9.7 cm)	0.44 (−0.08, 0.97)	−0.33 (−1.40, 0.73)
40–49	(8.2 cm)	1.20 (0.24, 2.15)	0.43 (−1.35, 2.20)	(10.3 cm)	0.71 (−0.19, 1.62)	0.16 (−1.71, 2.03)
50–59	(8.5 cm)	0.92 (−0.43, 2.28)	0.64 (−1.78, 3.06)	(11.3 cm)	0.21 (−1.24, 1.67)	−1.67 (−4.45, 1.11)
	Waist-Hip Ratio^b^			Waist-Hip Ratio^b^		
18–29	(0.066)	−0.05 (−0.48, 0.37)	−0.38 (−0.90, 0.14)	(0.065)	0.57 (0.21, 0.94)	0.37 (−0.04, 0.78)
30–39	(0.072)	0.19 (−0.35, 0.72)	−0.74 (−1.43, −0.05)	(0.071)	0.74 (0.22, 1.25)	0.59 (−0.02, 1.19)
40–49	(0.079)	0.75 (−0.15, 1.64)	−0.08 (−1.27, 1.12)	(0.078)	1.31 (0.44, 2.19)	1.22 (0.24, 2.19)
50–59	(0.084)	1.02 (−0.27, 2.30)	0.83 (−0.84, 2.49)	(0.083)	1.50 (0.06, 2.94)	1.43 (−0.16, 3.03)
	Waist-Height Ratio^b^			Waist-Height Ratio^b^		
18–29	(0.058)	−0.04 (−0.47, 0.39)	−1.53 (−2.43, −0.64)	(0.060)	0.62 (0.25, 0.98)	0.36 (−0.36, 1.08)
30–39	(0.067)	0.62 (0.08, 1.16)	−1.25 (−2.45, −0.06)	(0.075)	0.69 (0.18, 1.21)	0.77 (−0.34, 1.89)
40–49	(0.071)	1.09 (0.18, 2.00)	−0.07 (−2.24, 2.10)	(0.079)	1.23 (0.36, 2.10)	2.13 (0.49, 3.77)
50–59	(0.078)	1.41 (0.07, 2.74)	3.06 (0.15, 5.97)	(0.087)	1.41 (−0.04, 2.87)	3.01 (0.05, 5.97)

Estimates exclude those on antihypertensive medication. Model 1, estimates from multiple linear regression, adjusted for education and location. Model 2, model 1 plus additional adjustments for ^a^Waist circumference or ^b^Body mass index. *SD* standard deviation, *CI* confidence interval

**Table 6:** Mean differences in mean arterial pressure for anthropometric measures (*n* = 7 366)

		Male			Female	
		mmHg per SD (95% CI)			mmHg per SD (95% CI)	
		Model 1	Model 2		Model 1	Model 2
Age	Measurement (SD)			Measurement (SD)		
	Body Mass Index^a^			Body Mass Index^a^		
18–29	(3.6 kg/m^2^)	3.82 (3.39, 4.25)	1.88 (1.03, 2.72)	(4.0 kg/m^2^)	2.90 (2.47, 3.33)	2.31 (1.51, 3.11)
30–39	(4.2 kg/m^2^)	3.77 (3.08, 4.46)	1.15 (−0.25, 2.56)	(4.9 kg/m^2^)	4.99 (4.34, 5.65)	2.69 (1.34, 4.03)
40–49	(4.4 kg/m^2^)	4.55 (3.43, 5.67)	1.43 (−1.08, 3.93)	(5.3 kg/m^2^)	4.52 (3.58, 5.46)	0.51 (−1.20, 2.22)
50–59	(4.6 kg/m^2^)	3.79 (2.42, 5.15)	0.75 (−1.99, 3.49)	(5.6 kg/m^2^)	3.22 (1.94, 4.51)	0.90 (−1.53, 3.33)
	Waist Circumference^b^			Waist Circumference^b^		
18–29	(9.8 cm)	3.85 (3.42, 4.27)	2.24 (1.41, 3.08)	(9.2 cm)	2.61 (2.19, 3.04)	0.69 (−0.10, 1.48)
30–39	(11.8 cm)	3.96 (3.29, 4.64)	2.97 (1.57, 4.36)	(11.5 cm)	4.98 (4.33, 5.64)	2.63 (1.29, 3.97)
40–49	(12.4 cm)	4.77 (3.66, 5.89)	3.49 (0.99, 6.00)	(12.3 cm)	5.21 (4.29, 6.13)	4.78 (3.06, 6.50)
50–59	(13.4 cm)	4.16 (2.80, 5.52)	3.51 (0.76, 6.25)	(13.3 cm)	3.46 (2.20, 4.73)	2.70 (0.30, 5.11)
	Hip Circumference^b^			Hip Circumference^b^		
18–29	(7.3 cm)	3.76 (3.31, 4.20)	1.68 (0.92, 2.45)	(8.1 cm)	2.58 (2.14, 3.02)	0.54 (−0.23, 1.30)
30–39	(8.1 cm)	3.51 (2.81, 4.21)	1.07 (−0.20, 2.34)	(9.7 cm)	4.22 (3.55, 4.88)	−0.35 (−1.66, 0.96)
40–49	(8.2 cm)	3.92 (2.76, 5.08)	0.04 (−2.09, 2.17)	(10.3 cm)	4.21 (3.26, 5.16)	0.96 (−0.99, 2.92)
50–59	(8.5 cm)	4.01 (2.65, 5.37)	2.77 (0.34, 5.19)	(11.3 cm)	2.87 (1.62, 4.12)	0.77 (−1.61, 3.14)
	Waist-Hip Ratio^b^			Waist-Hip Ratio^b^		
18–29	(0.066)	2.53 (2.09, 2.96)	0.62 (0.11, 1.13)	(0.065)	1.53 (1.09, 1.96)	0.25 (−0.23, 0.72)
30–39	(0.072)	3.27 (2.61, 3.94)	1.63 (0.78, 2.48)	(0.071)	3.75 (3.08, 4.41)	1.66 (0.92, 2.40)
40–49	(0.079)	3.99 (2.92, 5.07)	2.00 (0.58, 3.42)	(0.078)	4.07 (3.14, 5.00)	2.63 (1.63, 3.63)
50–59	(0.084)	3.02 (1.72, 4.33)	1.24 (−0.43, 2.92)	(0.083)	2.37 (1.12, 3.62)	1.28 (−0.09, 2.64)
	Waist-Height Ratio^b^			Waist-Height Ratio^b^		
18–29	(0.058)	3.55 (3.12, 3.97)	1.18 (0.30, 2.05)	(0.060)	2.65 (2.23, 3.07)	0.84 (0.01, 1.67)
30–39	(0.067)	3.83 (3.17, 4.50)	2.81 (1.33, 4.28)	(0.075)	4.85 (4.21, 5.49)	2.50 (1.13, 3.86)
40–49	(0.071)	4.55 (3.46, 5.64)	3.00 (0.42, 5.58)	(0.079)	4.94 (4.05, 5.83)	4.33 (2.64, 6.02)
50–59	(0.078)	4.25 (2.92, 5.59)	4.40 (1.49, 7.31)	(0.087)	3.44 (2.20, 4.68)	2.97 (0.44, 5.49)

Estimates exclude those on antihypertensive medication. Model 1, estimates from multiple linear regression, adjusted for education and location. Model 2, model 1 plus additional adjustments for ^a^Waist circumference or ^b^Body mass index. *SD* standard deviation, *CI* confidence interval

**Table 7:** Mean differences in mid-blood pressure for anthropometric measures (*n* = 7 366)

		Male			Female	
		mmHg per SD (95% CI)			mmHg per SD (95% CI)	
		Model 1	Model 2		Model 1	Model 2
Age	Measurement (SD)			Measurement (SD)		
	Body Mass Index^a^			Body Mass Index^a^		
18–29	(3.6 kg/m^2^)	3.88 (3.43, 4.33)	2.02 (1.15, 2.89)	(4.0 kg/m^2^)	3.01 (2.56, 3.45)	2.35 (1.51, 3.18)
30–39	(4.2 kg/m^2^)	3.94 (3.22, 4.66)	1.47 (0.00, 2.94)	(4.9 kg/m^2^)	5.10 (4.40, 5.79)	2.67 (1.24, 4.09)
40–49	(4.4 kg/m^2^)	4.76 (3.54, 5.98)	1.60 (−1.12, 4.31)	(5.3 kg/m^2^)	4.65 (3.61, 5.68)	0.37 (−1.53, 2.26)
50–59	(4.6 kg/m^2^)	3.93 (2.43, 5.44)	0.68 (−2.34, 3.71)	(5.6 kg/m^2^)	3.36 (1.91, 4.81)	0.85 (−1.90, 3.60)
	Waist Circumference^b^			Waist Circumference^b^		
18–29	(9.8 cm)	3.88 (3.43, 4.32)	2.15 (1.28, 3.02)	(9.2 cm)	2.72 (2.28, 3.17)	0.77 (−0.05, 1.59)
30–39	(11.8 cm)	4.07 (3.36, 4.78)	2.80 (1.34, 4.26)	(11.5 cm)	5.11 (4.42, 5.80)	2.77 (1.35, 4.19)
40–49	(12.4 cm)	4.97 (3.76, 6.18)	3.54 (0.82, 6.26)	(12.3 cm)	5.41 (4.39, 6.43)	5.10 (3.20, 7.01)
50–59	(13.4 cm)	4.35 (2.85, 5.85)	3.75 (0.72, 6.79)	(13.3 cm)	3.64 (2.20, 5.07)	2.92 (0.20, 5.64)
	Hip Circumference^b^			Hip Circumference^b^		
18–29	(7.3 cm)	3.83 (3.36, 4.29)	1.73 (0.94, 2.52)	(8.1 cm)	2.66 (2.20, 3.12)	0.50 (−0.29, 1.30)
30–39	(8.1 cm)	3.66 (2.92, 4.39)	1.09 (−0.24, 2.42)	(9.7 cm)	4.29 (3.58, 5.00)	−0.41 (−1.79, 0.98)
40–49	(8.2 cm)	4.12 (2.87, 5.38)	0.11 (−2.19, 2.42)	(10.3 cm)	4.33 (3.28, 5.38)	0.99 (−1.17, 3.15)
50–59	(8.5 cm)	4.16 (2.66, 5.66)	2.88 (0.20, 5.55)	(11.3 cm)	2.90 (1.49, 4.32)	0.49 (−2.20, 3.18)
	Waist-Hip Ratio^b^			Waist-Hip Ratio^b^		
18–29	(0.066)	2.52 (2.06, 2.97)	0.56 (0.03, 1.08)	(0.065)	1.62 (1.17, 2.07)	0.31 (−0.18, 0.80)
30–39	(0.072)	3.30 (2.61, 4.00)	1.51 (0.61, 2.40)	(0.071)	3.87 (3.17, 4.57)	1.76 (0.98, 2.54)
40–49	(0.079)	4.12 (2.95, 5.28)	1.99 (0.45, 3.53)	(0.078)	4.29 (3.27, 5.32)	2.83 (1.72, 3.94)
50–59	(0.084)	3.19 (1.75, 4.63)	1.38 (−0.46, 3.23)	(0.083)	2.62 (1.21, 4.03)	1.51 (−0.03, 3.06)
	Waist-Height Ratio^b^			Waist-Height Ratio^b^		
18–29	(0.058)	3.54 (3.10, 3.98)	0.92 (0.02, 1.83)	(0.060)	2.76 (2.32, 3.19)	0.90 (0.04, 1.76)
30–39	(0.067)	3.94 (3.24, 4.63)	2.60 (1.05, 4.15)	(0.075)	4.96 (4.29, 5.64)	2.63 (1.18, 4.07)
40–49	(0.071)	4.73 (3.55, 5.92)	2.99 (0.19, 5.79)	(0.079)	5.15 (4.16, 6.13)	4.69 (2.82, 6.55)
50–59	(0.078)	4.49 (3.01, 5.96)	4.91 (1.70, 8.13)	(0.087)	3.68 (2.28, 5.08)	3.47 (0.61, 6.33)

Estimates exclude those on antihypertensive medication. Model 1, estimates from multiple linear regression, adjusted for education and location. Model 2, model 1 plus additional adjustments for ^a^Waist circumference or ^b^Body mass index. *SD* standard deviation, *CI* confidence interval

### Anthropometric measures and hypertension

**Fig. 1:**
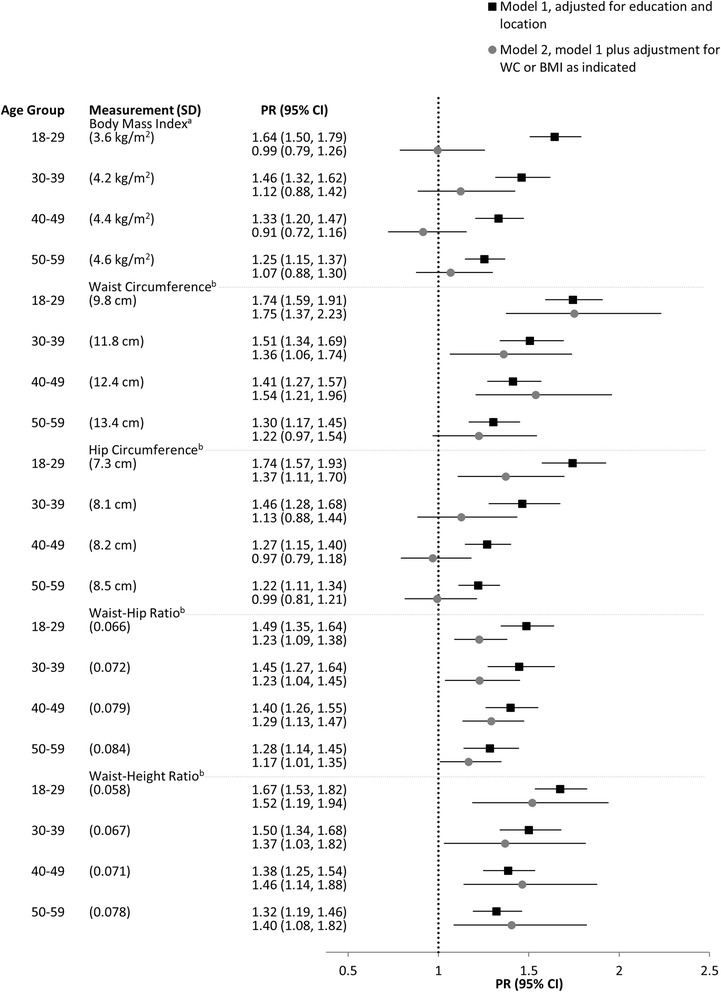
Prevalence ratios for hypertension per SD of each anthropometric measure among men (*n* = 3 746). Additional adjustment for ^a^WC or ^b^BMI. PR, prevalence ratio; WC, waist circumference; BMI, body mass index; SD, standard deviation; CI, confidence interval

**Fig. 2:**
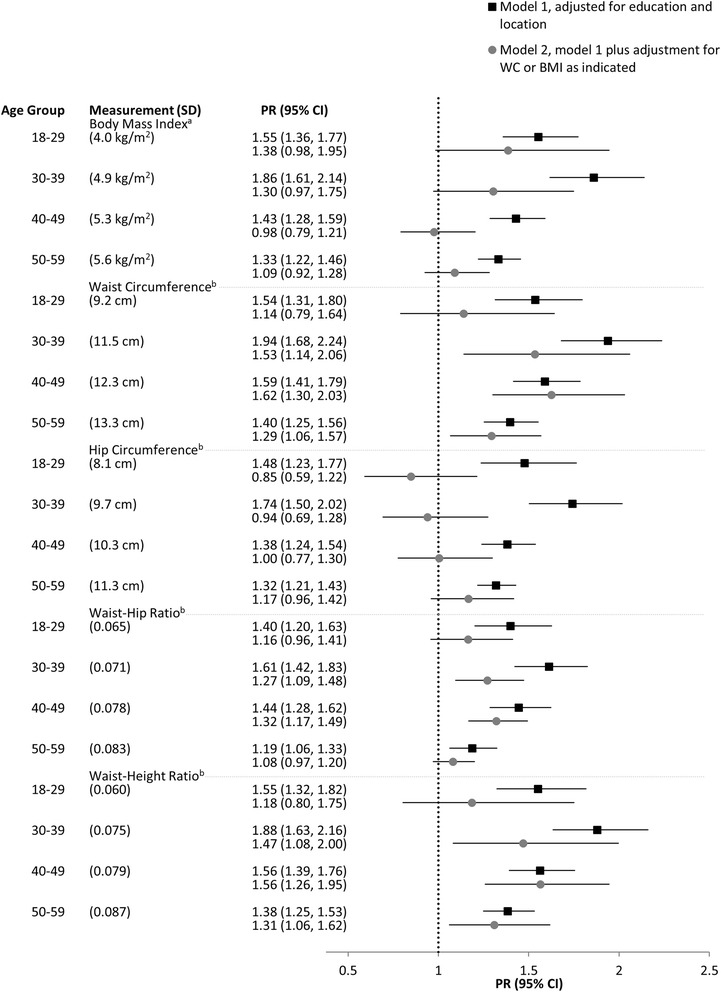
Prevalence ratios for hypertension per SD of each anthropometric measure among women (*n* = 3 855). Additional adjustment for ^a^WC or ^b^BMI. PR, prevalence ratio; WC, waist circumference; BMI, body mass index; SD, standard deviation; CI, confidence interval

Figures 1 and 2 show the PRs (95% CI) for hypertension according to age groups for men and women, respectively. Regardless of the anthropometric measure of adiposity, all were significantly (*p* < 0.05) associated with hypertension when considered separately (model 1). Overall, the associations of central adiposity measures (mainly WC or WHtR) with hypertension were slightly stronger than between BMI and hypertension for both sexes. The relationships of HC and WHR with hypertension were somewhat weaker than those observed with WC and WHtR.

After additional adjustment for WC, the relationship between BMI and hypertension was largely diminished across all age groups, particularly for men (model 2). Conversely, with a few exceptions, the relationship of WC and WHtR with hypertension remained predominantly unaffected after the additional adjustment for BMI. However, for women in the youngest (18–29 years) age group, additional adjustment for BMI reduced the association of WC and WHtR with hypertension. In addition, although not statistically significant (*p* = 0.37), there was a slight negative association between HC and hypertension for women in the youngest age group.

## Discussion

In our study, central adiposity, as assessed by WC was more strongly associated with the majority of blood pressure components and hypertension than general adiposity, as assessed by BMI. In addition, the relationship of WC with blood pressure and hypertension was largely independent of BMI. Our findings suggest that central adiposity may be a more important determinant of blood pressure and hypertension than general adiposity for adults in India.

Irrespective of the anthropometric measure of adiposity, when considered separately each was strongly and positively associated with SBP, DBP, and hypertension. Our results showed that, on average, every 5 kg/m^2^ greater BMI was associated with a 5 mmHg higher SBP and a 4 mmHg higher DBP, which is similar to results from a large study of a mainly European population [16]. However, it is well known that there are considerable differences in body fat distribution according to ethnicity. South Asians tend to have more abdominal adiposity when compared with Europeans [17]. Moreover, independent of sex, greater abdominal adiposity, whether visceral or subcutaneous is associated with many deleterious metabolic risk factors, including higher SBP, DBP and odds of hypertension [18]. Thus, anthropometric measures that capture central body fat distribution may be more informative for describing adverse health outcomes in a South Asian population.

With few exceptions, WC or WHtR was the measure with the strongest relation to nearly all blood pressure components independent of BMI. Among men in the younger (18–29 and 30–39 years) age groups, the relation between WC and SBP was somewhat weaker than between BMI and SBP. Nevertheless, in the same age groups, WC was more strongly associated with DBP than BMI. Indeed, DBP may be a more important component of blood pressure than SBP in these age groups; since many studies have shown the significance of DBP over SBP as the main driver of vascular risk in young adults [19]. Moreover, WC was also more strongly associated with mid-blood pressure than BMI for men across all age groups. This finding could be of particular importance, given that mid-blood pressure has been shown to be a more informative component than either SBP or DBP alone to predict vascular mortality [5].

Apart from the youngest (18–29 years) age group, the associations between anthropometric measures and blood pressure among women were similar to those for men. The observed difference for younger women may be explained, at least in part, by the marked heterogeneity in body fat distribution between men and women, and differences in the rate of abdominal fat accumulation. For instance, women generally have a higher percentage of body fat with a more peripheral distribution as compared to men, who have a more central distribution [20]. Hence, a measure of general adiposity, such as the BMI, may better reflect this peripheral fat distribution and be somewhat more strongly related to SBP and DBP than WC in young women. However, independent of body weight, an increase in abdominal adiposity occurs throughout life, with rates of gain being faster for men than women [21]. This difference in abdominal adiposity gain may provide an explanation for why the independent association of WC or WHtR with SBP and DBP was observed across all age groups for men, and not until older (≥30 years) age groups for women.

The associations of anthropometric measures with hypertension were, for the most part, comparable to those observed with continuous blood pressure. A slight negative (albeit not statistically significant) relationship was present between HC and hypertension after adjusting for BMI among women in the youngest age group. This may be due, in part, to the protective effects of an increased HC. A larger HC for a given BMI may be indicative of increased gluteofemoral fat, which has been shown to be independently associated with a better metabolic profile, and decreased odds of diabetes, dyslipidemia, and hypertension [22, 23]. Furthermore, it may influence blood pressure through direct effects on vascular health, whereby increased gluteofemoral fat is associated with lower arterial stiffness and aortic calcification [22].

Relative to the other anthropometric measures considered in our study, the association of WHR with all blood pressure components and hypertension was weak. The comparative differences in central body fat distribution may not be indicated by the WHR. For example, a similar WHR can be obtained by having both a large WC and HC or a small WC and HC. Moreover, differences in abdominal fat, specifically visceral adiposity may not be aptly reflected by changes in WHR [24]. This may explain, to some extent, the weaker associations found between WHR, blood pressure and hypertension. In comparison, after adjusting for BMI the association of WHtR with hypertension, although attenuated, was stronger than with HC and WHR for both sexes, especially for the oldest (50–59 years) age group. Indeed, our results suggest that WHtR may be of particular relevance in older (≥50 years of age) adults. On average, in later adulthood after peak height is reached, there is a reduction of height with age [25]. Additionally, it has been shown that adult height is negatively associated with SBP and pulse pressure, and the strength of these associations increases with age [26]. Thus, it is possible that the association between WHtR and hypertension may be augmented by the concomitant increases in abdominal adiposity, reflected by a larger WC, and decreases in height with age. Accordingly, when compared with the other anthropometric measures, WHtR was more strongly associated with both SBP and pulse pressure after the additional adjustment for BMI in the oldest age group. A stronger association between WHtR and pulse pressure among older adults may be of particular relevance, due to the relationship between pulse pressure and higher CVD risk in this subgroup [27, 28].

Several mechanisms that link greater adiposity to elevated blood pressure and risk of hypertension have been proposed [9, 10]. In light of our findings, mechanisms that relate general adiposity to blood pressure may be of particular relevance for young (<30 years of age) adult women. However, in general, our results suggest that blood pressure may predominantly be associated with abdominal adiposity in adults. Of these mechanisms, increased blood pressure may be the results of oxidative stress, inflammation or physical compression of the kidneys by excess abdominal adiposity, particularly visceral adiposity [9]. For instance, excess visceral and retroperitoneal fat, along with physical compression, may infiltrate the kidneys, leading to impaired pressure natriuresis and hypertension [9]. Nonetheless, further clarification of the potential sex and age related differences in mechanisms linking body fat distribution and blood pressure in India, and elsewhere are warranted.

Our study has some limitations that should be considered when interpreting the results. First, the cross-sectional design does not allow for causal inferences to be made about the relationships of adiposity with blood pressure and hypertension. Second, there may be other confounding factors than those available for consideration in our study. However, the largely causal association between adiposity and blood pressure is well established [8]. Thus, it can be speculated that excess adiposity precedes increased blood pressure and hypertension. Moreover, because of the predominantly causal relationship between adiposity and blood pressure, additional adjustment for other potential confounding factors may do little to alter these relationships. Indeed, additional adjustments for alcohol consumption and tobacco use did not materially affect the estimates or alter the observed associations. Third, although effort was made to obtain all circumference measurements on bare skin, some participants did not want to move aside their light clothing, which may be partly due to cultural reasons. Nevertheless, clothing was accounted for by subtracting 1 cm from measured values. Fourth, even though our study used measured blood pressure, it was only measured during one occasion. In comparison, the use of ambulatory blood pressure monitoring may provide better prognostic value [29]. Despite this, large studies have demonstrated the utility of blood pressure measurements obtained during a single occasion for the prediction of CVD risk [3–6]. Additionally, ambulatory blood pressure monitoring is not as feasible in LMICs, such as India. Lastly, no direct measure of body composition was done. Hence, it is important to keep in mind that although anthropometric measures correlate well with body fat; there is variation in body composition for a given value of any particular anthropometric measure of adiposity [21]. Notwithstanding these limitations, our findings provide guidance for future prospective studies of the relationships between adiposity, its distribution, and CVD risk in India. Further, our study is much larger than previous studies [11–14], and is a sample of the general population of men and women from different regions of India.

## Conclusion

In summary, we provide evidence for the discrete relationships between anthropometric measures of general and central adiposity with various blood pressure components and hypertension in India. Greater emphasis should be placed on measures of central adiposity, such as WC and WHtR for both sexes, than BMI as an adiposity measure in future studies investigating the associations between risk factors for high blood pressure and CVD outcomes.

## Abbreviations

BMI: Body mass index
CI: Confidence interval
CVD: Cardiovascular disease
DBP: Diastolic blood pressure
HC: Hip circumference
LMIC: Low and middle-income country
PR: Prevalence ratio
SBP: Systolic blood pressure
SD: Standard deviation
WC: Waist circumference
WHR: Waist-hip ratio
WHtR: Waist-height ratio
